# Supplementary material for: T-Cell-Dominated Immune Response Resolves Protracted SARS-CoV-2 Infection in the Absence of Neutralizing Antibodies in an Immunocompromised Individual
Source: Microorganisms. 2023 Jun 12;11(6):1562. doi: 10.3390/microorganisms11061562 (PMC10304262; doi:10.3390/microorganisms11061562)
Supplement: Supplementary file 1 [file microorganisms-11-01562-s001.zip › microorganisms-2406282-supplementary.pdf]

Supplementary Figure

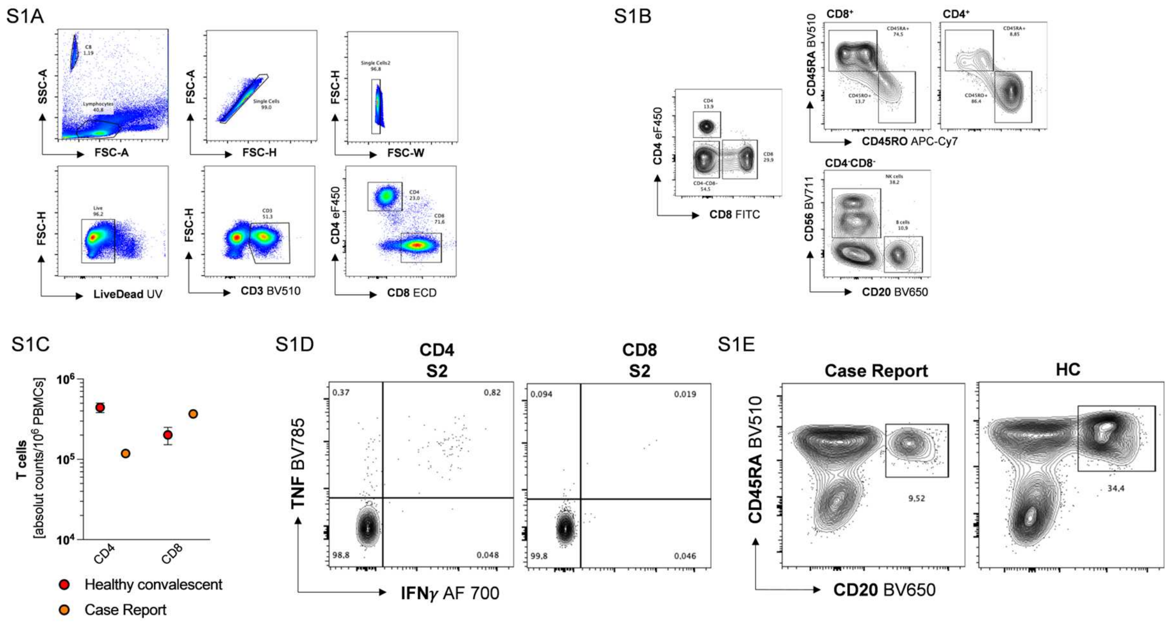

Supplementary Figure S1: Immune phenotyping demonstrates CD8 T cell-driven response. (a) Gating strategy used for intracellular flow cytometry **(b)** Gating strategy for phenotypic surface marker characterization **(c)** Absolute counts of CD4 and CD8 T cells per 10^6 PBMCs, in case report and non-immunocompromised convalescent (HC, n=9) individuals (Mean ±SD shown for HC group) **(d)** S2-reactive IFNγ and TNF-producing CD4 and CD8 T cells of the case report patient [7m after initial vaccination] determined directly ex vivo by flow cytometry using intracellular cytokine staining **(e)** Observed reduction in $CD20^+$ B cells in case report patient, as compared to a representative HC subject. Gated on $CD4^-$ $CD8^-$ live, singlets.
